# Tristetraprolin Posttranscriptionally Downregulates TRAIL Death Receptors

**DOI:** 10.3390/cells9081851

**Published:** 2020-08-07

**Authors:** Won Hyeok Lee, Myung Woul Han, Song Hee Kim, Daseul Seong, Jae Hee An, Hyo Won Chang, Sang Yoon Kim, Seong Who Kim, Jong Cheol Lee

**Affiliations:** 1 Department of Otorhinolaryngology, Ulsan University Hospital, University of Ulsan College of Medicine, Ulsan 44033, Korea; say1004s@gmail.com (W.H.L.); kongboribab@hanmail.net (M.W.H.); epoch55@naver.com (S.H.K.); iop5075@naver.com (D.S.); wogml3546@naver.com (J.H.A.); 2 Biomedical Research Center, Ulsan University Hospital, University of Ulsan College of Medicine, Ulsan 44033, Korea; 3 Department of Otolaryngology, Asan Medical Center, University of Ulsan College of Medicine, Seoul 05505, Korea; chyowon@hotmail.com (H.W.C.); sykim2@amc.seoul.kr (S.Y.K.); 4 Department of Biochemistry and Molecular Biology, Asan Medical Center, University of Ulsan College of Medicine, Seoul 05505, Korea; 5 Department of Otorhinolaryngology, GangNeung Asan Hospital, University of Ulsan College of Medicine, Gangneung 25440, Korea

**Keywords:** tumor necrosis factor-related apoptosis-inducing ligand, death receptor, tristetraprolin, posttranscriptional modification, AU-rich elements, cancer treatment

## Abstract

Tumor necrosis factor (TNF)-related apoptosis-inducing ligand (TRAIL) has attracted attention as a potential candidate for cancer therapy. However, many primary cancers are resistant to TRAIL, even when combined with standard chemotherapy. The mechanism of TRAIL resistance in cancer cells has not been fully elucidated. The TRAIL death receptor (DR) 3′-untranslated region (3′-UTR) is reported to contain AU-rich elements (AREs) that are important for regulating DR mRNA stability. However, the mechanisms by which DR mRNA stability is determined by its 3′-UTR are unknown. We demonstrate that tristetraprolin (TTP), an ARE-binding protein, has a critical function of regulating DR mRNA stability. DR4 mRNA contains three AREs and DR5 mRNA contains four AREs in 3′-UTR. TTP bound to all three AREs in DR4 and ARE3 in DR5 and enhanced decay of DR4/5 mRNA. TTP overexpression in colon cancer cells changed the TRAIL-sensitive cancer cells to TRAIL-resistant cells, and down-regulation of TTP increased TRAIL sensitivity via DR4/5 expression. Therefore, this study provides a molecular mechanism for enhanced levels of TRAIL DRs in cancer cells and a biological basis for posttranscriptional modification of TRAIL DRs. In addition, TTP status might be a biomarker for predicting TRAIL response when a TRAIL-based treatment is used for cancer.

## 1. Introduction

Tumor necrosis factor (TNF)-related apoptosis-inducing ligand (TRAIL), which was independently identified in both 1995 and 1996 and is also known as Apo-2 ligand (Apo2L), is a member of the TNF cytokine superfamily [1,2]. By binding to death receptor (DR) 4 or DR5, TRAIL induces tumor cell apoptosis without causing toxicity in normal cells. The cancer-specific action of TRAIL has attracted attention as a potential candidate for cancer therapy. Extensive preclinical studies conducted on recombinant human TRAIL (rhTRAIL) and TRAIL receptor agonists (TRAs) against TRAIL-receptors have demonstrated successful results with safety and tolerance even at high-dose administration to cancer patients. However, the outcomes of numerous clinical trials have failed to achieve beneficial anticancer activity because many primary cancers are resistant to TRAIL, even when combined with the standard chemotherapy [3,4]. TRAIL resistance mechanisms include transcriptional and posttranscriptional events controlling receptor expression, disrupted balance between anti-apoptotic and pro-apoptotic proteins, reduced caspase function, impaired death receptor signaling, short in vivo half-life of TRAIL proteins, low efficacy of TRAIL-based agents in their current forms or formulations, and off-target toxicity [4,5]. The mechanism underlying the heterogeneity of surface expression of DR has been widely investigated; previous studies reported epigenetic silencing [6,7,8], deficient surface transport of DR4 [9], mutations resulting in DR4 and DR5 loss-of-function [10,11,12,13], and posttranslational modifications of synthesized receptor proteins [14]. Posttranscriptional regulation has not been previously established as a cause of the down-regulation of DR expression in cancer cells.

Posttranscriptional regulation is the key aspect of gene expression regulation, and 3′-untranslated regions (3′-UTRs) are closely related to posttranscriptional regulation [15]. AU-rich elements (AREs) are AUUUA-destabilizing motifs located in the 3’-UTR of a variety of short-lived mRNAs that mediate expression of many cytokines and proto-oncogenes [16]. The stability of ARE-containing mRNAs is regulated by numerous ARE-binding proteins. Tristetraprolin (TTP), encoded by *ZFG36* gene, is one of the most characterized ARE-binding proteins that promotes degradation of ARE-containing transcripts [17]. TTP contains a cysteine–cysteine-cysteine–histidine (CCCH) zinc finger motif that recognizes cis-acting AREs in the 3′-UTR of target mRNA. After initial high-affinity binding to AREs in a sequence- and structure-specific manner, TTP generally facilitates mRNA decay by recruiting enzymes for the rapid shortening of the poly(A) tail [18]. Since its discovery as a regulator of tumor necrosis factor mRNA stability more than 20 years ago, TTP has become notable for its mediation of multiple cancers and immunity-associated transcripts. In malignant tumors, cumulative evidence has shown that TTP has tumor-suppressive properties and loss of TTP expression or function was closely related to tumor onset and progression and was associated with poor outcomes in patients with various cancers. TTP has become more obvious in regulating mRNA targets at various molecular cancer traits; cell cycle regulators, angiogenesis, apoptosis, pro-tumorigenic inflammatory mediators, cellular senescence, epithelial-mesenchymal transition, and metastasis [19]

In the present study, we found that DR4/5 contains multiple copies of AREs in its 3′-UTR and investigated for the first time the role of TTP in posttranscriptional regulation of TRAIL DR4/5 gene expression in human colon cancer cells. Overexpression of TTP decreased DR4/5 expression levels in cancer cells, and TTP promoted degradation of DR4/5 mRNA by binding to the 3′-UTR of mRNA. Overexpression of TTP in colon cancer cells decreased DR4/5 synthesis and significantly inhibited TRAIL-induced apoptosis of colon cancer cells, while transfection of DR4/5 cDNA without the 3′-UTR restored TRAIL sensitivity. Our data demonstrated that DR4/5 mRNA contains several AREs within the 3′-UTR and TTP operates its destabilizing activity by directly binding DR4/5 AREs. These results establish DR4/5 mRNA as a physiologic target of TTP and suggest that TTP regulation by DR4/5 transcript stability may be a key biomarker for the expression of DR4/5 observed in human cancers.

## 2. Materials and Methods

### 2.1. Cell Culture

Four human colorectal cancer cell lines, HCT116, KM12C, HT29, and SW480, were purchased from the Korean Cell Line Bank (Korean Cell Line Research Foundation, Seoul, Korea) and maintained in RPMI1640 supplemented with 10% fetal bovine serum (Gibco BRL/Life Technologies, Inc., Gaithersburg, MD, USA). They were cultured at 37 °C in a humidified chamber containing 5% CO_2_. Human recombinant TRAIL purchased from Sigma were used at indicated concentrations.

### 2.2. Viability Assay

At the indicated times, CellTiter 96^®^ Aqueous One Solution Reagent (Promega, Madison, WI, USA) was added to each well according to the manufacturer’s instructions, and absorbance at 490 nm (OD490) was determined for each well using a Wallac Vector 1420 Multilabel Counter (EG&G Wallac, Turku, Finland).

### 2.3. Semi-Quantitative RT-PCR and Quantitative Real-Time PCR (qRT-PCR) Analysis

Five micrograms of DNase I-treated total RNA were reverse transcribed using oligo-dT and Superscript II reverse transcriptase (Invitrogen, Carlsbad, CA, USA) according to the manufacturer’s instructions. Semi-quantitative RT-PCR was performed using Taq polymerase (SolGent, Daejeon, Korea) and the appropriate primers: DR4 forward, 5′-GGG ACA GCA CGG ACC CAGTG-3′ and reverse, 5′-ATC CTT GAC CTT GAC CATCC-3′; DR5 forward, 5′-GCG GTC CTG CTG TTG GTCTC-3′ and reverse, 5′-GCT TCT GTC CAC ACG CTCAG-3′; TTP forward, 5′-CGC TAC AAG ACT GAG CTAT-3′ and reverse, 5′-GAG GTA GAA CTT GTG ACAGA-3′; GAPDH forward, 5′-ACA TCA AGA AGG TGG TGAAG-3′ and reverse, 5′-CTG TTG CTG TAG CCA AATTC-3′.

For qRT-PCR, Total RNA was isolated using a PureLink™ RNA Mini Kit (Thermo Fisher Scientific, Waltham, MA, USA), and cDNA was subsequently synthesized using a first-strand cDNA synthesis kit and by reverse transcription-polymerase chain reaction (iNtRON Biotechnology, Seoul, Korea). SYBR^®^ Green master mix (Applied Biosystems, Foster City, CA, USA) was used for qRT-PCR with a PRISM^®^ 7500 sequence detection system (Applied Biosystems). All reactions were performed in triplicate in 96-well plates, and the mean values were used to calculate the mRNA expression. The primer sequences were as follows: TTP forward, 5′-CGC TAC AAG ACT GAG CTAT-3′ and reverse, 5′-GAG GTA GAA CTT GTG ACAGA-3′; DR4 forward, 5′-AGC CTG TCA TCT GTG GGATT-3′ and reverse, 5′-CTC AAG TAC ACA CTC CAA AG-3′; DR5 forward, 5′-CTT TGT GGC CTT CTT TGAAG-3′ and reverse, 5′-CCA CAC AGT TGC TCC ACAT-3′; GAPDH forward, 5′-ACA TCA AGA AGG TGG TGAAG-3′ and reverse, 5′-CTG TTG CTG TAG CCA AATTC-3′.

### 2.4. Quantitative Real-Time PCR (qRT-PCR) Analysis for RNA Kinetics

For RNA kinetic analysis, we used actinomycin D and assessed DR4 and DR5 mRNA expression by quantitative PCR. Total RNA was isolated using a PureLink™ RNA Mini Kit (Thermo Fisher Scientific, Waltham, MA, USA), and cDNA was subsequently synthesized using a first-strand cDNA synthesis kit by reverse transcription-polymerase chain reaction (iNtRON Biotechnology, Seoul, Korea). SYBR^®^ Green master mix (Applied Biosystems, Foster City, CA, USA) was used for qRT-PCR with a PRISM^®^ 7500 sequence detection system (Applied Biosystems). All reactions were performed in triplicate in 96-well plates, and the mean values were used to calculate mRNA expression. The primer sequences were as follows: DR4 forward, 5′-AGC CTG TCA TCT GTG GGA TT-3′ and reverse, 5′-CTC AAG TAC ACA CTC CAA AG-3′; DR5 forward, 5′-CTT TGT GGC CTT CTT TGAAG-3′ and reverse, 5′-CCA CAC AGT TGC TCC ACAT-3′; GAPDH forward, 5′-ACA TCA AGA AGG TGG TGAAG-3′ and reverse, 5′-CTG TTG CTG TAG CCA AATTC-3′.

### 2.5. Plasmid, siRNAs, Transfection, and Dual-Luciferase Assay

SW480 cells that overexpressed human TTP were generated using the pcDNA6/V5 vector (Invitrogen, Carlsbad, CA, USA). Full-length human cDNA of TTP was cloned by RT-PCR from the RNA of KM12C cells using forward primer 5′-CCG TGA ATT CAT GGA TCT GAC TGC CAT-3′ and the reverse primer 5′-CAC TCT CGA GCT CAG AAA CAG AGA TGC-3′. The product was subcloned into the pcDNA6/V5 vector. Roughly 1.5 × 10^7^ cells were electrophorated with 20 μg of pcDNA6/V5-TTP at 500 V, 975 lF with a Gene Pulser electroporator II (Bio-Rad Laboratories, Hercules, CA, USA). After transfection, SW480/ pcDNA6/V5-TTP cells stably transfected with human TTP were selected by adding Blasticidin (10 ug of Blasticidin/mL; Invitrogen) 3 days after transfection. Stable polyclonal transfectants were maintained in bulk culture without further clonal purification. Stable polyclonal transfectants were tested for overexpression of human TTP by RT-PCR and Western blots using anti-human TTP polyclonal antibody (ab33058; Abcam, Cambridge, UK). A control cell line, SW480/pcDNA6/V5, was generated by transfection with the pcDNA6/V5 vector. KM12C was transfected with TTP-siRNA (sc-36760; Santa Cruz, Dallas, TX, USA) or control siRNA-A (sc-37007; Santa Cruz) using LipofectamineTM RNAiMAX (Invitrogen). Cells were seeded in six-well plates at a concentration of 3 × 105 cells/mL. The concentration of siRNA was 45 nM. Cells were harvested 24 h after transfection. Typically, cells were analyzed for loss of TTP mRNA and protein expression 24 h after transfection using either RT-PCR or immunoblotting, respectively. A variety of deletion mutants of the DR4 and DR5 3′-UTR were PCR-amplified from cDNA of SW480 cells using Taq polymerase and primer sets as follows: DR4 Frag-ARE-1, CCG CTC GAG TCC AAT AAG TCC CAT TTC ATA and ATA AGA ATG CGG CCG CAC TCA AGG TAA TAA ATT. DR5 Frag-ARE-1-4, CCG CTC GAG CCT AAT GTA AAT GCT and ATA AGA ATG CGG CCG CAA TTT GGTC; Frag-ARE-1, CCG CTC GAG CCT AAT GTA AAT GCT and ATA AGA ATG CGG CCG CGT TCA TATC; Frag-ARE-1/2, CCG CTC GAG CCT AAT GTA AAT GCT and ATA AGA ATG CGG CCG CCT CAT ATGT; Frag-ARE-1-3, CCG CTC GAG CCT AAT GTA AAT GCT and ATA AGA ATG CGG CCG CCC AAA AACT. PCR products were inserted into the XhoI/NotI sites of a psiCHECK2 Renilla/firefly Dual-Luciferase expression vector (Promega, Madison, WI, USA). Mutant oligonucleotides in which AUUUA pentamers were substituted with AGCA were also synthesized. The oligonucleotides were ligated into the XhoI/NotI sites of the psiCHECK2 vector. For luciferase assays, SW480 cells were co-transfected with various psiCHECK-DR4 and DR5 3′-UTR constructs and pcDNA6/V5-TTP using TurboFectTM in vitro transfection reagent. Transfected cells were lysed with lysis buffer (Promega) and mixed with luciferase assay reagent (Promega), and the chemiluminescent signal was measured in a Wallac Victor 1420 multilabel counter. Firefly luciferase was normalized to Renilla luciferase in each sample. All luciferase assays reported here represent at least three independent experiments, each consisting of three wells per transfection.

### 2.6. RNA EMSA

The biotinylated RNA probes for wild type (wtDR4-EMSA, 5′-GCU AAG GUU AAU UUA UUA AUU AUU UAA UUU AUU UAC CUUG-3′, wtDR5-EMSA, 5′-TAG TCA TGG AAG GAT CAT TTA TGC AGG TAG TCA TTC CAGG-3′) and mutant (mutDR4-EMSA, 5′-GCT AAG GTT AAG CAT TAA TTA GCA AGC AGC ACC TTGA-3′, mutDR5-EMSA, 5′-TAG TCA TGG AAG GAT CAG CAT GCA GGT AGT CAT TCC AGG-3′) were synthesized by BIONEER Co. (Daejeon, Korea). RNA EMSA was conducted using Lightshift^TM^ Chemiluminescent EMSA Kit (Thermo Fisher Scientific, Waltham, MA, USA) according to the manufacturer’s instructions. Briefly, 20 fmol of biotinylated RNA was combined with 5 μg of cytoplasmic protein of SW480/pcDNA6/V5-TTP cells in a binding buffer. For super-shift assays, the anti-TTP antibody (ab33058; Abcam, Cambridge, UK) or control antibody (I-5381; Sigma-Aldrich, St. Louis, MO, USA) were added to the reaction mixtures. After adding antibodies, reaction mixtures were incubated overnight on ice. The reaction mixtures were resolved on 5% native polyacrylamide gels in 0.5 Tris borate-EDTA (TBE) buffer. Gels were transferred to nylon N+ membranes in 0.5 × TBE at 400 mA and 4 °C for 1 h. RNAs were cross-linked to the membranes and detected by streptavidin-horseradish peroxidase binding and chemiluminescent detection.

### 2.7. Western Blot Analysis

Total protein was extracted using RIPA buffer containing proteases and phosphatase inhibitors (Thermo Fisher Scientific, Waltham, MA, USA), and protein concentration was determined with the Bradford protein assay kit (Bio-Rad Laboratories, Hercules, CA, USA). Proteins were separated by electrophoresis on a 10–13% SDS-polyacrylamide gel and transferred to a nitrocellulose membrane (Amersham International, Little Chalfont, UK). Membranes were blocked with 5% bovine serum albumin (BSA; bioWORLD, Dublin, OH, USA) in Tris-buffered saline with tween^®^ 20 (TBST) for 1 h at room temperature. Membranes were subsequently washed with Tris-buffered saline with tween^®^ 20 detergent (TBST) and incubated with primary antibodies to tristetraprolin (ab33058; Abcam, Cambridge, UK), DR4 (ab8414; Abcam), DR5 (ab8416; Abcam), and β-actin (sc-47778; Sigma-Aldrich, St. Louis, MO, USA), and diluted in 5% BSA/TBST overnight at 4 °C. Membranes were washed with TBST. The secondary antibody (anti-mouse or anti-rabbit IgG HRP conjugate; Bethyl Laboratories, Montgomery, TX, USA) was diluted 2000-fold in TBST and applied to cells for 1 h. After washing with TBST, specific binding of antibodies was detected using an ECL kit (Thermo Fisher Scientific, Waltham, MA, USA) following the manufacturer’s protocol.

### 2.8. Apoptosis by Annexin V/PI Analysis

Human colon adenocarcinoma cells were seeded on a 60 mm dish, incubated with TRAIL (30 ng/mL) for 24 h, washed twice with ice-cold PBS (pH 7.0), and incubated with fresh medium for 5 days. Senescence-induced cells were washed twice with ice-cold PBS (pH 7.0) and then resuspended in binding Buffer (500 μL). FITC-Annexin V 5 μL was added to 5 μL PI followed by incubation for 15 min at RT in the dark. Samples were analyzed using a fluorescence-activated flow cytometer (FACScan; Becton Dickinson, Franklin Lakes, NJ, USA).

### 2.9. In Vivo Antitumor Activity

Either SW480/pcDNA6/V5 or SW480/pcDNA6/V5-TTP (2 × 10^7^ cells) were injected into the flanks of 6-week-old nude (nu/nu) mice (Orient Bio Inc., Seongnam, Korea). Prior to treatment with TRAIL, tumor size was measured two to three times per week until the volume reached approximately 200 mm^3^. Tumor volume was calculated as W^2^ × L × 0.52, where L is the largest diameter and W is the diameter perpendicular to L. After establishing tumor xenografts, mice were randomized into four groups of five mice per group. Mice were fed ad libitum and maintained in environments with a controlled temperature of 22–24 °C and 12 h light and dark cycles. Mice in each treatment group were treated with TRAIL at a dose of 200 ng/kg by intra-tumoral injection twice per week for two weeks. All animal experimental procedures were approved by the Institutional Animal Care and Use Committee of the University of Ulsan Laboratory Animal Research Center. All animal experiments were performed in accordance with Institutional Animal Care and Use Committee (IACUC) guidelines. Ethical code number: 0118-06 (C1-0), date of approval: 6 December 2017.

### 2.10. Statistical Analysis

All statistical analyses and calculations were performed using Microsoft Excel spreadsheets and GraphPad Prism v.5 (GraphPad Software, San Diego, CA, USA). Group differences were determined with Student’s *t*-test or Mann–Whitney U test. Data are expressed as mean and standard deviation. All statistical tests were two-sided, and *p* values less than 0.05 were considered statistically significant.

## 3. Results

### 3.1. DR4/5 Expression is Inversely Correlated with TTP Expression in Human Colon Cancer Cell Lines

**Figure 1:**
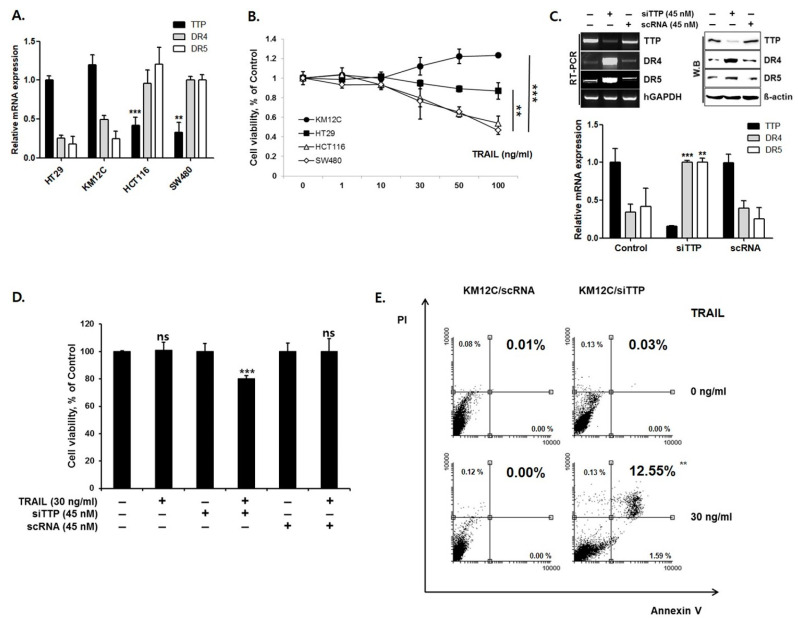
TTP inhibits DR4/5 expression in human colon cancer cells. (**A**) Levels of TTP, DR4, and DR5 expression in four human colon cancer cell lines were determined by quantitative real-time PCR (qRT-PCR). (**B**) MTS assay; viability of KM12C and HT29 cells with TRAIL treatment was higher than that of HCT116 and SW480 cells. KM12C cells with high TTP expression and low DR4/5 expression, and SW480 cells with low TTP expression and high DR4/5 expression were selected for further studies. (**C**) Expression of TTP and DR4/5 in TTP siRNA-treated or control siRNA (scRNA)-treated KM12C cells by qRT-PCR and Western blot analysis. (**D**) MTS assay; siTTP sensitizes KN12C to TRAIL. (**E**) FACS analysis. Late apoptosis and cell death in KM12C with siTTP increased significantly. ** *p* < 0.01; *** *p* < 0.001.

To determine whether DR4/5 expression is inversely correlated with endogenous TTP expression, the expression of DR4/5 and TTP was analyzed in four human colon cancer cell lines: HT29, KM12C, HCT116, and SW480. Cell lines with high TTP expression levels (HT29 and KM12C) exhibited low expression levels of DR4/5, and those with low TTP expression levels (HCT116 and SW480) showed relatively high DR4/5 expression levels (Figure 1A). These results suggest an inverse correlation between TTP expression and DR4/5 expression in human colon cancer cell lines. To determine differences in TRAIL sensitivity among cell lines, all cell lines were treated with TRAIL. An MTS assay showed that KM12C and HT29 cells were significantly more viable than HCT116 and SW480 cells after exposure to TRAIL (Figure 1B).

To test whether down-regulation of TTP affects DR4/5 expression, siRNA against TTP was used to reduce the expression level of TTP in KM12C cells. Down-regulation of TTP by treatment with siRNA significantly increased the expression level of DR4/5 (*p* < 0.01) (Figure 1C). However, treatment with control siRNA (scRNA) did not decrease the expression level of endogenous TTP and did not change DR4/5 expression on qRT-PCR and Western blot assay. An MTS assay in KM12C cells treated with siTTP showed increased sensitivity to TRAIL-mediated apoptosis (Figure 1D). Annexin V-FITC/PI staining, performed to confirm the cell viability measured by MTS assay, demonstrated that late apoptosis and cell death were significantly higher (12.55%, *p* < 0.01) in KM12C with siTTP cells than in KM12C with scRNA cells (Figure 1E).

**Figure 2:**
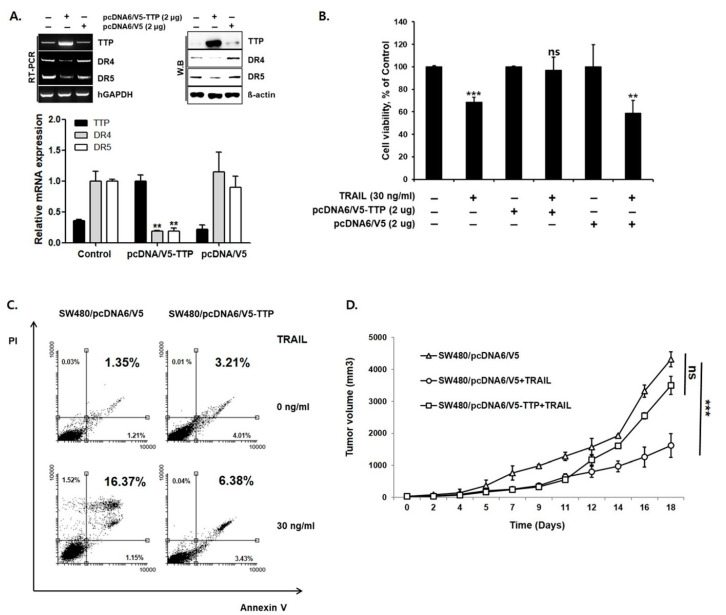
Overexpression of TTP reduced DR4/5 expression. (**A**) Expression of TTP and DR4/5 in SW480/pcDNA and SW480/TTP was determined by quantitative real-time PCR and Western blot analysis. MTS assay (**B**) and annexin V-FITC/PI staining assay (**C**) in TTP-overexpressed SW480/TTP show significant resistance to TRAIL treatment. (**D**) In vivo antitumor activity of TRAIL in TTP-overexpressed SW480 cells. When dorsal flank tumors were approximately 200 mm^3^, mice in each treatment group were treated with intratumoral injection of TRAIL (0.2 mg/kg twice per week for two weeks). The Control group received only normal saline. TTP overexpression in SW480/TTP cells shows significant TRAIL resistance. ** *p* < 0.01; *** *p* < 0.001.

We next examined whether overexpression of TTP reduces DR4/5 expression. For this purpose, SW480 cells with low TTP expression and high DR4/5 expression were selected. A TTP expression vector (pcDNA6/V5-TTP) was transfected into SW480 cells. As a negative control, SW480 cells were transiently transfected with the pcDNA6/V5 empty vector. Overexpression of TTP in pcDNA6/V5-TTP transfected SW480 cells was confirmed by qRT-PCR and Western blot analysis. Expression of DR4/5 was significantly reduced in pcDNA6/V5-TTP transfected SW480 cells compared to empty vector-transfected cells (*p* < 0.01) (Figure 2A). An MTS assay and annexin V-FITC/PI staining assay in TTP-overexpressed SW480 cells showed reduced sensitivity to TRAIL-mediated apoptosis (16.37% vs. 6.38%, *p* < 0.01) (Figure 2B,C). In a 20-day in vivo study, the average tumor volume in mice with TTP-overexpressing SW480 cells increased significantly under TRAIL treatment. TTP overexpression changed TRAIL sensitive SW480 cells to TRAIL-resistant SW480 cells (Figure 2D). Taken together, these results indicate that the TTP expression mediates TRAIL sensitivity via modulation of DR4/5 expression.

### 3.2. TTP Destabilized DR4/5 mRNA

**Figure 3:**
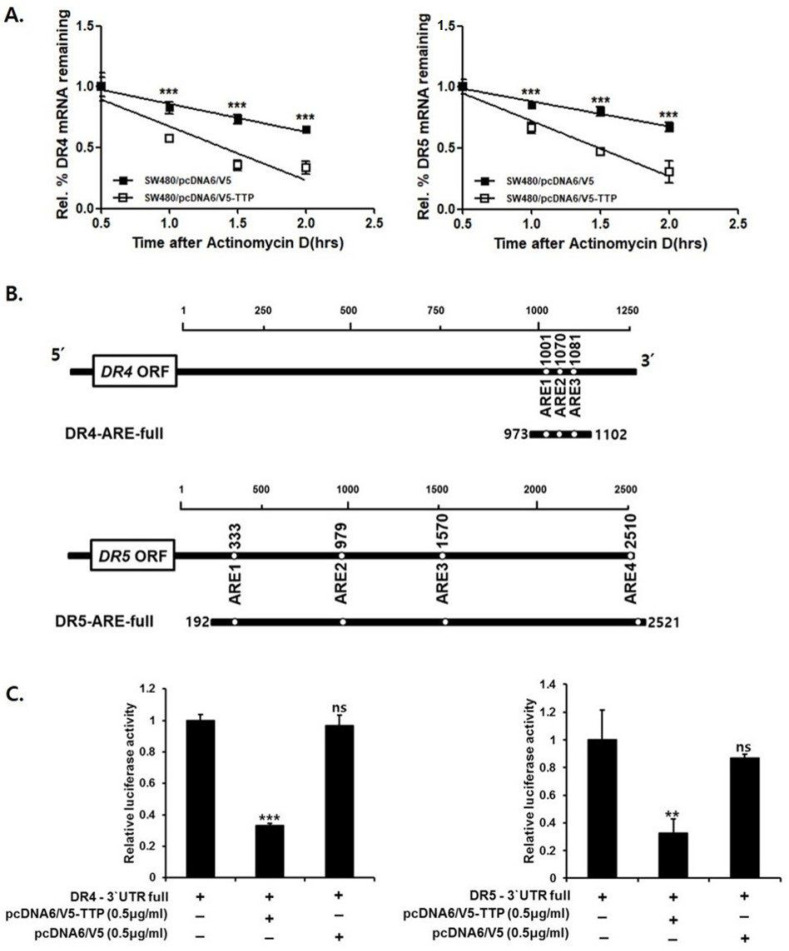
TTP destabilizes mRNA via interaction with ARE within DR4/5 mRNA 3′-UTR. (**A**) Expression of DR4 and DR5 mRNA in SW480/pcDNA and SW480/TTP cells was determined by quantitative real-time PCR at indicated times after adding 5 µg/mL actinomycin D. mRNA half-life was calculated by nonlinear regression of mRNA levels at indicated times after addition of actinomycin D. Results shown represent mean ± SD of three independent experiments (*** *p* < 0.001). (**B**) Analysis of full base pairs of DR4/5 3′-UTR. Schematic representation of luciferase reporter constructs used in this study. Oligonucleotides derived from full-bp DR4/5 mRNA 3′-UTR were cloned downstream of the luciferase reporter gene in psiCHECK luciferase expression vector. White circles represent wild-type pentameric motif AUUUA. (**C**) Inhibition of luciferase reporter containing DR4/5 mRNA 3′-UTR by TTP overexpression. SW480 cells were co-transfected with 500 ng of psiCHECK luciferase reporter construct containing full AREs in DR4/5 and pcDNA6/V5-TTP or empty vector pcDNA6/V5. After normalization, luciferase activity from SW480 cells transfected with psiCHECK vector alone was set to 1.0. Results represent the mean ± SD of three independent experiments (** *p* < 0.01; *** *p* < 0.001). ORF, open reading frame; ns, not significant.

To determine whether decreased expression of DR4/5 resulted from changes in the stability of DR4/5 mRNA, the half-life of this mRNA was measured by quantitative real-time PCR in pcDNA6/V5-TTP transfected SW480 cells and empty vector-transfected cells. The half-life of DR4/5 mRNA in empty vector-transfected cells was more than 2 h. However, in TTP-overexpressed SW480 cells, the half-life of DR4/5 mRNA was less than 1.5 h after actinomycin D treatment. These results indicate that increased expression of TTP contributes to decreased DR 4/5 levels through the destabilization of DR4/5 mRNA (Figure 3A). 

TTP protein regulates mRNA stability by binding to AREs within the mRNA 3′-UTR [17]. Analysis of the full base pair of DR4/5 3′-UTR revealed the presence of three AUUUA ARE motifs in DR4 and four ARE motifs in DR5 (Figure 3B). A luciferase reporter gene linked to the full DR4/5 3′-UTR containing all three AREs was used in the psiCHECK plasmid to determine whether downregulation of DR4/5 expression by TTP was mediated through the DR4/5 mRNA 3′-UTR. SW480 cells were co-transfected with 500 ng of psiCHECK luciferase reporter construct containing full AREs in DR4/5 and pcDNA6/V5-TTP or empty vector pcDNA6/V5. When SW-480 cells were transfected with a plasmid overexpressing TTP, luciferase activity from the full DR4/5 3′-UTR was significantly inhibited (Figure 3C).

### 3.3. TTP Binds to All Three ARE in DR4 and Only the 3rd ARE in DR5 mRNA 3′-UTR

**Figure 4:**
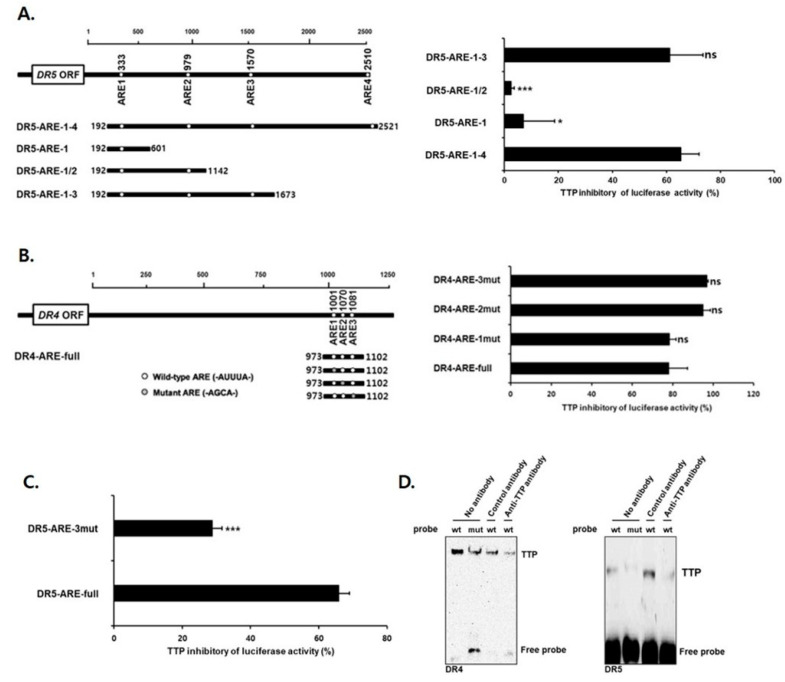
ARE3 within DR5 and all three AREs in DR4 mRNA 3′-UTR are essential for the inhibitory effect of TTP. Schematic representation of luciferase reporter constructs with (**A**) 2521-bp DR5 and (**B**) 1250-bp DR4 mRNA 3′-UTR used in this study. White circles represent wild-type pentameric motif AUUUA, and gray circles represent mutated (mut) motif AGCA. (**A**) Only fragments with 3rd ARE within the DR5 3′-UTR show TTP-mediated inhibition of DR5. (**B**) Each single mutant of DR4-ARE showed a similar TTP inhibitory effect. (**C**) A single mutant of 3rd ARE of DR5 was confirmed to prevent the TTP inhibitory effect. Fragments derived from 2521-bp DR5 and 1250-bp DR4 mRNA 3′-UTR were cloned downstream of the luciferase reporter gene in psiCHECK luciferase expression vector. Inhibition of luciferase reporter containing DR5 and DR4 3′-UTR by TTP overexpression. Mapping of sequence in DR5 and DR4 mRNA 3′-UTR required for TTP inhibition of luciferase activity. SW480 cells were co-transfected with 500 ng of psiCHECK luciferase reporter construct containing fragmented or full AREs in DR4/5 and pcDNA6/V5-TTP or empty vector pcDNA6/V5. TTP-induced inhibition of luciferase activity observed with each construct was compared to that obtained with empty vector pcDNA6/V5. Cells were harvested, and luciferase activity was normalized to firefly activity. Luciferase values obtained from cells transfected only with luciferase construct full AREs were set to 1. Results represent the mean ± SD of three independent experiments (* *p* < 0.05; *** *p* < 0.001). ORF, open reading frame; ns, not significant. (**D**) RNA EMSA; RNA EMSA was performed by mixing cytoplasmic extracts containing 4 µg of total protein from pcDNA6/V5-TTP-transfected SW480 cells with 20 fmol of biotinylated wild-type (wt) or mutant (mut) probe. Control antibody or anti-TTP was added to reaction mixtures. Binding reactions were then separated by electrophoresis on 5% polyacrylamide gel under nondenaturing conditions. TTP indicate the position of the TTP-containing band.

The next goal was to determine regions within the DR4/5 3′-UTR that are important for the TTP inhibitory effect. Luciferase genes linked to various deletion mutants of the DR5 3′-UTR were used (Figure 4A, left panel). Whereas TTP decreased the luciferase activity of the luciferase reporter gene cloned DR5-ARE-1-4 (containing all four AREs) by 65.3%, DR5 3′-UTR fragments DR5-ARE-1/2 (containing ARE1 and ARE2) and DR5-ARE-1 (containing ARE1) abrogated the inhibitory effect of TTP on luciferase activity (2.5% and 7.0% inhibition, respectively) (Figure 4A, right panel). However, the DR5 3′-UTR fragments DR5-ARE-1-3 (containing ARE1, ARE2, and ARE3) responded similarly to TTP compared with the whole DR5-ARE-1-4 construct, suggesting that ARE3 within the DR5 3′-UTR is responsible for the inhibitory effect of TTP.

Because of the close location of all three AREs in DR4, single mutants of each ARE motif were prepared to determine which ARE is responsible for the response to TTP (Figure 4B, left panel). Each single mutant of DR4-ARE showed a similar TTP inhibitory effect on reporter gene activity compared with DR4-ARE-full (Figure 4B, right panel), suggesting that all three AREs are involved in TTP binding and its inhibitory activity. A single ARE3 mutant in DR5 was confirmed to prevent the TTP inhibitory effect (Figure 4C). Although these results were obtained using ectopically overexpressed TTP protein, the significance of DR4 AREs and DR5 ARE3 for TTP binding was demonstrated.

To demonstrate the association between endogenous TTP and ARE in DR4/5 3′-UTR, RNA EMSA was conducted using a biotinylated RNA probe containing wild-type or mutant ARE in DR4/5. Cytoplasmic extracts were prepared from SW480 cells transfected with pcDNA6/V5-TTP to overexpress TTP and were incubated with the biotinylated RNA probe containing wild-type or mutant ARE3 in DR4/5. When the wild-type DR4 ARE3 probe was mixed with cytoplasmic extracts from TTP-overexpressed SW480, a dominant probe-protein complex was observed (Figure 4D, left panel). However, the mutant DR4 ARE3 probe failed to form this complex. The formation of the DR4 ARE3 probe-protein complex was reduced by preincubation of the reaction mixture with anti-TTP antibody but not with the control antibody. This result was also demonstrated in DR5, where the probe-protein complex was reduced compared to DR4. Collectively, these data strongly suggest that the expression of DR4/5 occurs through direct interaction of TTP with ARE3 of DR4/5 mRNA.

## 4. Discussion

The role of TTP as a key factor in posttranscriptional gene regulation has been established, in malignant tumors, TTP participates extensively in gene regulatory networks for tumor suppression, including oncogenes and cancer-related cytokines [19]. In this study, we focused TTP on DR expression and demonstrated TTP bound to the ARE motif of DR4/5 mRNA and enhanced the decay of DR4/5 transcripts. An inverse correlation was demonstrated between TTP and DR expression. TTP overexpression in cancer cells changed TRAIL-sensitive cancer cells to TRAIL-resistant cells, and down-regulation of TTP increased TRAIL sensitivity via restoration of DR4/5 expression. Our results suggest that TTP is a key negative regulator of DR4/5 expression and inhibition of TTP led to increased DR4/5 levels in cancer cells. These results, combined with the finding that a wide variety of cancer cells generally express high levels of cytoplasmic DRs, and TTP expression is significantly suppressed in many cancer cells, might be strong evidence for the dynamic expression of DRs [20,21,22].

Dynamic expression of TRAIL DRs is one of the most widely investigated mechanisms for TRAIL-based therapy resistance because lack of surface DRs is sufficient to render cancer cells resistant to TRAIL-induced apoptosis regardless of the status of other apoptosis signaling mechanisms. However, in functional aspects of DRs until now, two issues need more investigation.

First, although many transcriptional factors and posttranslational modulations have been revealed to involve in DR4/5 expression, mRNA expression of DRs does not necessarily reflect their functional protein expression, and there is no correlation between total receptor protein expression levels and the sensitivity of tumors to TRAIL-based treatment [14]. Previous studies have demonstrated a variety of transcriptional factors such as p53, CHOP, NF-kB, FOXO3a, and AP1, as well as posttranslational regulations, which include protein glycosylation, trafficking, endocytosis processes, and autophagy, have been revealed to be associated with dynamic expression of DRs, [14,23,24]. Our results, the first report as far as we know, suggest a posttranscriptional modification of TTP may be another mechanism for modulation DR expression.

Second, besides their canonical locations in the plasma membrane and in intracellular membranes of the secretory pathway as well as endosomes and lysosome, the biologic relevance of noncanonical intracellular compartmentalization of DRs needs to be further defined. Previous numerous studies to validate the highly expressed cytoplasmic DR as an independent prognostic marker showed conflicting results in various cancers [25,26,27]. After a connection of the nuclear DRs with an apoptosis resistance became to know [28], DR expression in the nucleus or the cytoplasm was demonstrated to be associated with an unfavorable prognosis, therefore, it was suggested that DR-based risk stratification should be interpreted on their intracellular compartmentalization with consideration of co-expression of other TRAIL receptors in addition to DR4/5 [27,29]. Additional studies showed that DRs in noncanonical intracellular locations are likely to neither contribute to canonical apoptosis signaling nor to non-apoptotic signal transduction regardless of whether they are soluble within cytosol, trapped within the trans-Golgi network, in autophagosomes or in the nucleus. More recently, nuclear DR5 was discovered to result in increased levels of malignancy-promoting factors HMGA2 and Lin28B and enhanced tumor cell proliferation in vitro and in vivo [30]. Similarly, cytoplasmic DR4/5 was revealed to induce cell death in response to unresolved ER stress [31,32]. With regard to TTP in this specific function of noncanonical location of DRs, at what stage occur remains unknown, TTP may also act on DRs mRNA that are involved in these pathways because TTP regulate mRNA targets at various stages during carcinogenesis and modulate tumor cell apoptosis by directly regulating the apoptotic mediators within both intrinsic and extrinsic pathways [33].

In this study, the analysis of the full base pair of DR4/5 mRNA 3′-UTR revealed the presence of three ARE motifs in DR4 and four ARE motifs in DR5. Analyses using mutant ARE substrates indicate that that TTP binds to all three ARE in DR4, and only the 3rd ARE in DR5 mRNA 3′-UTR. This affinity difference may be originated from the fact that AREs are grouped into three classes based on the number and distribution of AUUUA pentamers, and that TTP is reported to have a different binding affinity for the three classes. Thermodynamic basis permitting high-affinity RNA recognition was established in RNA substrate specificity by the RNA-binding domain of TTP [34]. Various other explanations, including prior occupancy of the AREs by protective proteins, were also suggested for the mechanisms of gene and cell-type specificity of mRNA decay [35].

As induced DRs can easily bind to TRAIL to induce apoptosis, there is considerable interest in increasing DR expression with clinically used or developing anticancer compounds. TTP may be another novel molecular target for therapeutic intervention that improves the sensitivity of TRAIL-induced apoptosis by increasing expression of TRAIL or its death receptors. However, the safety of altered TTP concentration must be carefully considered for clinical applications [36]. Because tumor necrosis factor (TNF) alpha mRNA stability was first established TTP target, TRAIL is the member of TNF superfamily, and TRAIL also had AREs in its mRNA 3′-UTR, additional work will be required for the role of TTP in the expression of TRAIL as well as other death receptors besides DR4/5.

In conclusion, we demonstrated that TTP is important for the posttranscriptional regulation of DR4/5 gene expression. As a result, TTP-induced downregulation of death receptors led to decreased TRAIL-induced apoptosis. This study provides a molecular mechanism for the enhanced levels of TRAIL DRs in cancer cells. TTP-mediated enhancement of DR mRNA degradation expands our understanding of the regulation of TRAIL DR expression in cancer cells. Additional work is required to obtain an in-depth understanding of TTP in cancer biology, especially in the regulation of TRAIL and prior to the clinical use of TRAIL as a new therapeutic agent for cancer treatment. However, this study provides a biological basis for post-transcriptional modification of TRAIL DRs and may provide a novel strategy for predicting and restoring cancer cell sensitivity to TRAIL-induced apoptosis. In addition, TTP status may be a biomarker for predicting TRAIL response when a TRAIL-based cancer treatment is used.
